# Extent of surgery in clinically evident but operable MTC – when is central and/or lateral lympadenectomy indicated?

**DOI:** 10.1186/1756-6614-6-S1-S3

**Published:** 2013-03-14

**Authors:** Oliver Gimm

**Affiliations:** 1 Department of Clinical and Experimental Medicine, Faculty of Health Sciences, Linköping University, SE-58185 Linköping, Sweden; 2 Department of Surgery, County Council of Östergötland, SE-58185 Linköping, Sweden

## Abstract

Medullary thyroid carcinoma (MTC) metastasizes very early lymphogeneously. It has been shown that the presence of lymph node metastases is associated with a worse outcome. Postoperative biochemical cure, i.e. normalization of posttherapeutical calcitonin levels, has been shown to correlate with a better outcome. The rate of biochemical cure decreases dramatically in the presence of lymph node metastases but can still be achieved in about 30-40% of patients despite the presence of lymph node metastases.

In 2009, the American Thyroid Association (ATA) published guidelines on the management of MTC. Various recommendations in the guidelines are dealing with the extent of lymph node dissection in different clinical settings. This article summarizes and comments on these recommendations.

## Background

Medullary thyroid carcinoma (MTC) metastasizes very early lymphogenously.

The presence of initial lymph node metastases in non-screening patients is generally reported to be higher than 50%, often as high as 75-80% [1,2]. Even bilateral lateral lymph node involvement is quite frequent (>25%) [2] and has been shown to be associated with distant metastases [3]. In these advanced cases, biochemical cure, i.e. normalization of posttherapeutical calcitonin levels, is extremely rare [4].

It has been shown that the presence of lymph node metastases in general is associated with a worse outcome [5]. Interestingly, it has less clearly been shown that lymphadenectomy improves the outcome in patients with MTC [5]. What has been shown is that, depending on patient selection, biochemical cure can be achieved in about 30-40% of patients with regional lymph node metastases [4,6] which, however, is a significantly lower rate than that for patients without lymph node metastases (90-100%) [1,6]. Biochemical cure itself has been shown to correlate with a better outcome [7-9] but late recurrence has been shown.

That the extent of lymph node dissection is part of ongoing discussion has apart from the unknown benefit in a specific patient in part to do with the possible complications that are accompanied with this procedure, mainly hypoparathyroidism and nerve injury [10]. In addition, in the presence of advanced disease, t.ex. distant metastases, the benefit of lymph node dissection is even more questionable.

## ATA guidelines regarding lymphadenectomy

The guidelines published by the American Thyroid Association (ATA) [11] contain various recommendations dealing with the extent of lymphadenectomy.

### Clinically evident or highly suspected MTC

According to the guidelines, patients having MTC or those where MTC is highly likely should undergo total thyroidectomy and prophylactic central compartment (level VI) neck dissection both with and without evidence of cervical lymph node metastases in the absence of advanced local invasion and distant metastases (recommendation 61 & 62). The lateral compartment (level II-V) should be included in the case of ultrasound-confirmed lateral lymph node metastases (recommendation 63). Preoperative ultrasound is therefore indicated in all patients prior to surgery (recommendation 19).

Some members of the task force recommended prophylactic inclusion of the lateral compartment recommended in the presence of central compartment lymph node metastases (recommendation 62). This recommendation is based on the finding that the involvement of the ipsilateral cervicolateral compartment correlates with the number of lymph node metastases in the central compartment [12].

### Advanced disease

In general, a less aggressive approach is advised in the presence of advanced local and/or distant metastases while maintaining locoregional disease control to prevent central neck morbidity (recommendation 64 & 65). In patients with distant metastases, removal of small asymptomatic and nonthreatening lymph node metastases (<1 cm) is of unknown benefit and such lymph nodes may be observed (recommendation 81).

### Primary incomplete surgery

Following primary incomplete surgery, reoperation including the central lymph node compartment is recommended when neck US is suspicious for persistent local disease in the central or lateral neck compartment (recommendation 71) or when the basal serum calcitonin level is above the normal reference range (recommendation 72).

If not previously performed, node dissection in both the central and lateral compartment should be compartment-oriented. Removal of only grossly metastatic lymph nodes, or other limited procedures, should be avoided in these instances (recommendation 77). This recommendation is based on the fact that removal of only grossly metastatic lymph nodes correlates with a lower calcitonin normalization rate, a higher reoperation rate and a lower survival rate [13].

In view of the ATA guidelines, central lymph node dissection remains controversial if primary surgery only consisted of total thyroidectomy in patients with continuously elevated calcitonin levels in the absence of radiographic findings (recommendation 78). It is explained in the guidelines that controversy also exist due to the increased risk of harm. It is obvious that the surgical experience and expertise plays an important role in this regard. Of note, it must be primarily assumed that continuously elevated calcitonin levels following thyroidectomy mainly are due to lymph node metastases left *in situ*. Calcitonin normalization has in selected patients been observed in up to 35-40% even after reoperation [14,15] and may actually be as low as 6% if one waits for radiographic positive findings [16]. Thus the recommendation to wait for positive imaging as suggested by the ATA guidelines is questionable.

### Hereditary MTC

In patients with hereditary MTC, early identification of patients, e.g. through family screening, may enable to limit the extent of surgery to the thyroid gland since lymph node metastases may be very unlikely if preoperative calcitonin levels are normal [17,18]. Besides patient’s age, primary tumor size and calcitonin levels may be helpful in determining the surgical extent. The ATA guidelines give some recommendations in this regard (recommendations 33, 35, 40-43). Since this review is focusing on clinically evident MTC, these recommendations will not be discussed in detail here.

## Further comments

In general, lymph node dissection should be very restrictive in the presence of advanced local and/or distant disease. This is justified by the very low chance to cure those patients [6] where no benefit might be gained from the lymph node dissection. In these instances, the calcitonin doubling-times may be of help assessing patient’s prognosis where doubling-tomes less than 6 months are very unfavourable [19].

### Clinically evident or highly suspected lymph node metastases

Taken the above into account, dissection is almost always indicated in the presence of cervical lymph nodes metastases. Clinically, the presence of lymph node metastases is most often assumed in the presence of enlarged lymph nodes. Fine-needle aspiration is rarely performed. However, lymph node metastases in MTC are typically rather small in size and, thus, normal lymph node size does not exclude the presence of metastases.

### Unknown lymph node status

If no enlarged lymph nodes are present, the extent of lymph node dissection is much more difficult to assess. This situation, however, is very common.

Most surgeons would recommend central lymphadenectomy (defined as dissection of level VI) in all patients with sporadic MTC and in all screening patients with elevated calcitonin levels at the time thyroidectomy is performed due to the high likelhood of the presence of positive lymph nodes [20]. The exception are screening patients with normal calcitonin levels that probably have an extremely low risk that lymph node metastases exist [17,18]. These patients may therefore forego lymphadenectomy. As mentioned before, one reason to avoid cervical lymphadenectomy is the observed higher rate of complications [10]. The reason to perform central lymphadenectomy synchronous with thyroidectomy is that metachronous lymphadenectomy confers an even higher risk of complications. This does not apply to the lateral compartments (level II-V) that are not touched if the previous operation consist of thyroidectomy both with and without central lymphadenectomy.

Since the ipsilateral cervicolateral compartment contains almost as often lymph node metastases as the central compartment [21], some surgeons would recommend performing both central and lateral lymphadenactomy synchronous with thyroidectomy. Others have recommended lateral lymph node dissection “on demand”, i.e. in the setting of measurable postoperative bCT and/or sCT levels indicating LN metastasis [22]. In the case of contralateral positive lymph nodes, the chance of biochemical cure decreases dramatically [21] and no beneficial outcome has been shown if lymphadenectomy is performed in these patients.

### Assessing the lymph node status

The question is how the extent of lymph node metastases can be assessed pre- or at the latest intraoperatively in MTC patients.

#### Imaging techniques

The accuracy of computed tomography and ultrasonography for evaluating cervical lymph node metastases in patients with papillary thyroid carcinoma is not very high, 74% vs. 68 resp. [23]. Since it is known that lymph mode metastases in MTC are often rather small as compared to papillary thyroid carcinoma, the accuracy is expected to be lower which has indeed been shown [24]. Both, somatostatin receptor scintigraphy and, in particular, FDG-PET have been used with some success in detecting lymph node metastaes preoperatively but the reported series are small [24-28].

In reoperative cases, a variety of imaging techniques (ultrasound, computed tomography, FDG-PET) seems to have a low sensitivity (56%, 42%, and 32%, respectively).

Thus, currently available imaging techniques are of minor value identifying small lymph node metastases. Ultrasound, which is readily available, is the investigation of choice and should always be performed first.

#### Venous sampling

In reoperative cases, it has been shown that pentagastrin-stimulated intravenous calcitonin sampling followed by targeted surgery may be beneficial in the diagnostic work-up of MTC after thyroidectomy [29]. However, the number of reported cases is low and the real value is not known yet.

#### Primary tumor size

Even small tumors (<1 cm) inherit a risk for lymph node metastases that increases with tumor size [30]. Patients having a primary tumor of less than 5 mm in size have a risk of having lymph node metastases between 13-20% if sporadic and between 6-14% if hereditary. The biochemical cure rates at these early stages are 69-85% and 80-96%, respectively. Concerning tumors smaller than 1 cm in size, lymph node metastases are found in 5-31% and the biochemical cure ranges from 71% to 100% (reviewed in [30]). Thus, even at these early stages, lymph node metastases are not uncommon [31].

Thus, lymph node metastases are not uncommon even in small primary tumors. The tumor size is, hence, of less value in assessing the presence of lymph node metastases in patients with MTC.

#### Lymph node metastases in the central compartment

Studies have shown that the involvement of the ipsilateral cervicolateral compartment correlates with the number of lymph node metastases in the central compartment. If more than 3 lymph nodes in the cervicocentral compartment were involved, almost all patients had lateral lymph node metastases in one study [12]. This “marker”, however, is of less use in the clinical setting in the absence of macroscopically enlarged lymph nodes since it is not possible to determine the numbers of central lymph node metastases within a decent time period intraoperatively so far. Of note, lateral lymph node metastases were found in about 10% even in the absence of central lymph node metastases. This finding, i.e. the presence of cervicolateral lymph node metastases without cervicocentral compartment involvement, often referred to as skip metastases [32], has even been found in up to 35% by others [33].

#### Calcitonin levels

Concerning microMTC (<1 cm), calcitonin levels cannot distinguish between patients with and without lymph node metastases [22]. This is also true for patients with larger primary tumors even though higher basal calcitonin levels correlate better than pentagastrin stimulated calcitonin levels with lymph node involvement [20]. In the latter study, both central and ipsilateral lateral lymph node metastases were present in about 10% of the patients already at a basal level of 20 pg/mL. In patients higher than 200 pg/mL, contralateral lateral lymph node involvement was observed in at least 12% of the patients.

One study showed that intraoperative stimulated calcitonin levels following thyroidectomy and central lymph node dissection correlated well with the presence of lateral lymph node metastases [34]. The intraoperative usefulness of this technique would require quick calcitonin assays. Currently, the analysis takes several hours.

#### Age

While age plays an important role assessing timing and extent in patients with hereditary MTC [35], it does not seem to play a role in sporadic MTC.

#### Other “markers”

Beside these more traditional markers, other markers are sought after.

Of interest, one such “marker” has been cervical pain. In one study, the authors reported that roughly 80% of their patients with MTC presented with neck pain (defined as any subjective complaint of anterior neck discomfort, ache, pressure, or sharp, throbbing, or dull sensations in the region of the thyroid gland, with or without palpation) as opposed to only 6% of their patients with papillary thyroid carcinoma [36]. However, even 36% of those without pain had lymph node metastases.

Desmoplastic stromal reaction is one of those intraoperative markers that appear to have more clinical value. In one study analyzing 120 MTCs, it was shown that desmoplastic stromal reaction, which can be assessed intraoperatively, has a low (38%) sensitivity of predicting N0 but a very high (100%) specificity. The authors therefore recommend avoiding lymph node dissection in the absence of desmoplastic stromal reaction [37]. The results where later shown to be reproducible by other pathologists [38] but larger prospective studies are missing yet.

## Conclusions

If advanced disease is absent, central lymph node dissection is almost always indicated at the initial operation in patients with clinically evident MTC. Synchronous inclusion of the ipsilateral lateral compartment is recommended by some but might probably also be done ”on demand” if postoperative calcitonin levels remain elevated.

In reoperative cases, the extent of lymph node dissection depends primarily on the extent of previous operation(s). Compartments that have not been operated on in a systematic way, i.e. dissection of all lymph node and the connective tissue *“en bloc”*, should be operated in this manner. Node picking should be reserved to those compartments that have been operated on systematically. I these cases, positive imaging is required. This approach, however, has not been proven to be of any benefit for the patient.

Future research has to concentrate on improving preoperative imaging and on the identification of intraoperative measurable markers that correlate with the presence or absence of lymph node metastases with a high accuracy.

## Competing interests

The author declares no competing interests.

## Declarations

This article has been published as part of *Thyroid Research* Volume 6 Supplement 1, 2013: European comments on Medullary Thyroid Cancer Management Guidelines of the American Thyroid Association. The full contents of the supplement are available online at http://www.thyroidresearchjournal.com/supplements/6/S1. Publication of this supplement has been supported by the European Thyroid Association-Cancer Research Network.
